# Emory Caregiver Research Interest Group: Advancing Dementia Caregiver Research for Scholars

**DOI:** 10.1093/geroni/igaf122.1509

**Published:** 2025-12-31

**Authors:** Maria Quiñones-Cordero, Zachary Baker, Emily Mroz, Kalisha Bonds Johnson, Kenneth Hepburn, Fayron Epps

**Affiliations:** University of Rochester School of Nursing, Rochester, New York, United States; Arizona State University, Phoenix, Arizona, United States; Emory University, Atlanta, Georgia, United States; Emory University, Atlanta, Georgia, United States; Emory University, Atlanta, Georgia, United States; UT Health San Antonio, San Antonio, Texas, United States

## Abstract

Dementia caregiving occurs within diverse contexts, so there are ongoing opportunities to develop and test behavioral interventions to promote context-specific caregiver mastery. The NIA-supported Emory Roybal Center for Dementia Caregiving Mastery established the Caregiver Research Interest Group (CRIG) to advance behavioral intervention research in untargeted caregiving contexts and to support the career development of early-stage investigators nationwide. CRIG convenes monthly virtual discussions to identify research gaps, generate innovative intervention strategies, and foster interdisciplinary collaboration. These meetings serve as an incubator for novel intervention concepts, a forum for refining research methodologies, and a networking platform that facilitates cross-disciplinary engagement. By exploring the intersections between diverse caregiving environments and behavioral intervention research, scholars refine research questions that address the unique challenges faced by dementia caregivers. CRIG also provides vital career advancement opportunities, including discussions with National Institute on Aging Program Officers on funding priorities, exposure to training programs for early-career investigators, and individualized consultations on research trajectories. Compared to other programs in this symposium CRIG is particularly flexible, possessing less formal targeted outcomes. CRIG includes 21 scholars (post-doctoral through professor). Feedback from scholars will be discussed in this session including highlighted accomplishments (i.e., collaborations in publications and abstracts; leadership and group feedback on grant proposals leading to successful funding) and areas of improvement such as increasing diversity in expertise and fostering more peer collaborations. Through leadership-driven engagement, CRIG fosters a dynamic research community dedicated to enhancing dementia caregiver mastery science and expanding the impact of intervention research in diverse caregiving contexts.

